# Weighted Correlation Network Analysis (WGCNA) Applied to the Tomato Fruit Metabolome

**DOI:** 10.1371/journal.pone.0026683

**Published:** 2011-10-21

**Authors:** Matthew V. DiLeo, Gary D. Strahan, Meghan den Bakker, Owen A. Hoekenga

**Affiliations:** 1 Boyce Thompson Institute for Plant Research, Ithaca, New York, United States of America; 2 Robert W. Holley Center for Agriculture and Health, Agricultural Research Service (ARS), United States Department of Agriculture (USDA), Ithaca, New York, United States of America; 3 Eastern Regional Research Center, Agricultural Research Service (ARS), United States Department of Agriculture (USDA), Wyndmoor, Pennsylvania, United States of America; Semmelweis University, Hungary

## Abstract

**Background:**

Advances in “omics” technologies have revolutionized the collection of biological data. A matching revolution in our understanding of biological systems, however, will only be realized when similar advances are made in informatic analysis of the resulting “big data.” Here, we compare the capabilities of three conventional and novel statistical approaches to summarize and decipher the tomato metabolome.

**Methodology:**

Principal component analysis (PCA), batch learning self-organizing maps (BL-SOM) and weighted gene co-expression network analysis (WGCNA) were applied to a multivariate NMR dataset collected from developmentally staged tomato fruits belonging to several genotypes. While PCA and BL-SOM are appropriate and commonly used methods, WGCNA holds several advantages in the analysis of highly multivariate, complex data.

**Conclusions:**

PCA separated the two major genetic backgrounds (AC and NC), but provided little further information. Both BL-SOM and WGCNA clustered metabolites by expression, but WGCNA additionally defined “modules” of co-expressed metabolites explicitly and provided additional network statistics that described the systems properties of the tomato metabolic network. Our first application of WGCNA to tomato metabolomics data identified three major modules of metabolites that were associated with ripening-related traits and genetic background.

## Introduction

The technologies common to systems biology approaches – transcriptomics, proteomics, ionomics and metabolomics (the “-omics”) – are now capable of generating data orders of magnitude more efficiently than was previously possible. This increasingly economical flood of data is placing very significant limitations on the ability of scientists to store, process and analyze it [1], [2]. While many field are grappling with the contemporary challenge of “big data”, it is proving particularly demanding for many biologists, who historically have often been able to rely on relatively simple statistical and computational methods. Although such well-known methods (*e.g.* ANOVA) are powerful when investigating small numbers of variables, most are extremely susceptible to the multiple testing problem in large systems biology datasets, often forcing the user to invoke biology-independent filters (such as minimum expression fold change thresholds) in order to produce manageable numbers of interesting candidates and to limit Type I and Type II errors [3]. Thus, new approaches are needed that are, ideally, both efficient in extracting meaningful associations from these highly multivariate datasets and are easily and intuitively understood by the end user.

Network analyses have been proposed as a solution to systems biology studies, particularly those involving transcriptomic datasets, as this approach both models the interactions of real biological networks and is intuitively understood by users [4], [5], [6]. The reconstruction of biological networks allows processes to be examined from a truly systems-scale perspective and provides unique insight into the structure and behavior of the molecular interactions that underlie important phenomena such as development and nutrient deficiency stress [7], [8]. Furthermore, the clustering of co-expressed molecules into "modules" mirrors regulatory associations found in biological systems and provides information on unknown nodes through "guilt by association" with well-characterized ones. Such analyses can be focused on identifying properties associated with key molecules or can be applied in a non-targeted manner, where the networks themselves are the primary focus of interest. The latter approach often leads to the interrogation of molecules that display certain network properties or associations with external traits [4]. Although numerous improved methods have been proposed to model and describe these networks, it is not difficult to find studies that continue to rely on more traditional, and less effective, approaches to analyze such highly complex datasets.

Weighted gene correlation network analysis (WGCNA) is one new approach to network modeling that relies on easily understood statistical methods and improves on simple correlation networks [9]. WGCNA has been implemented in R, a free and open source statistical programming language that is widely used, maintained and improved by an active community [10]. WGCNA was developed to more efficiently analyze microarray datasets by quantifying not only the correlations between individual pairs of genes, but also the extent to which these genes share the same neighbors. The resulting topological overlap matrix is then converted to a dissimilarity measure and submitted to hierarchical clustering. This process creates a dendrogram that clusters similarly expressed genes into discrete branches, with the most highly connected nodal points or "hubs" located at the branch tips. WGCNA, as implemented in R, then provides various methods through which individual branches can be clustered in separate "modules" [9]. By allowing additional genetic, phenotypic, developmental and behavioral traits to be associated with tens of modules or nodal points, instead of thousands of individual variables (*i.e.* gene probes), WGCNA not only alleviates the multiple testing problem, but also provides a direct route through which the effects of experimental treatments can be detected in modeled networks. While WGCNA was developed to analyze transcriptomic profiling experiments, we assert that this method is equally powerful and illustrative to analyze metabolomic fingerprinting experiments.

Metabolomic technologies, which have historically lagged behind transcriptomics and proteomics, are proving increasingly useful in systems biology [11]. As a major component of economically important phenotypes such as quality and composition, the metabolome holds concrete relevance to the biology of organisms. Furthermore, as metabolites occupy a generally downstream location relative to the transcriptome and proteome, their presence is both likely to integrate the full sum of changes that occur in upstream regulatory steps and may additionally provide causal anchors to model directional biological networks [12], [13], [14]. Metabolomics additionally incurs relatively low per analysis costs relative to common transcriptomic and proteomic approaches [14]. Moreover, metabolomics can be taken advantage of to provide a high-throughput platform for phenotyping, which has become an increasingly unavoidable bottleneck in systems biology as upstream genomic, transcriptomic and proteomic technologies improve [15]. Although identification of detected metabolites is not trivial, a given molecular species, once elucidated, can be expected to have similar phenomenology in different organisms, which greatly assists the annotation of modules.

Metabolomic approaches are particularly valuable in plant biology, where more than 200,000 secondary metabolites have been identified so far [16]. This chemical diversity exists both within and among different chemical classes. For example, flavonoids currently contain more than 7,000 known members. This includes 232 different glycosides of a single flavonol, quercetin, one of the most common flavonoids in tomato fruit [17], [18], [19]. The chemical diversity found within tomato fruits in particular has been relatively well characterized and shown to play critical roles in physiology, development and ecology [13], [20], [21], [22], [23], [24], [25]. The tomato fruit metabolome varies not only with genetic background and due to the impact different alleles of key regulatory genes, but also by specific tissue type within the fruit. Furthermore, major metabolomic reorganizations are known to occur during the transition from green mature, through red ripe to overripe tomato fruit. The accumulation and dissolution of major primary and secondary metabolites has been well characterized, with dramatic changes in pigmentation, flavor, scent and nutritional quality due to variation in many compounds [13], [20], [22], [26], [27], [28], [29], [30].

Following initial pulses of cell division and expansion, a developing tomato fruit reaches the mature, green stage, at which point it becomes competent to undergo ripening. Initiation of tomato fruit ripening is climacteric, requiring a burst of respiration that is triggered by the phytohormone ethylene [20]. Tomato ripening is additionally regulated by a light-dependent pathway, which appears most critical for pigment accumulation (*e.g.* carotenoids and flavonoids) [20], [31]. Onset of ripening is additionally coordinated through transcription factors produced by genes such as *LeMADS-RIN*, which potentiates ethylene dependent fruit ripening. If *LeMADS-RIN* function is impaired, fruit development essentially arrests at the mature green stage [32], [33]. A spontaneous mutant form of *LeMADS-RIN* exists that exhibits a dose-dependent phenotype [33]. In the heterozygous state, this natural *rin* mutation inhibits ripening only partially, producing a fruit with an extended shelf life. Such *Rin/rin* genotypes have been used extensively in commercial fresh market tomatoes for decades [34].

Here, we apply non-targeted NMR profiling and WGCNA to explore the impact of the *rin* mutation on the tomato fruit metabolome, using the spontaneous mutation in two genetic backgrounds together with wild type tomatoes and a transgenic line expressing *LeMADS-RIN* in an antisense orientation. We additionally compare the relative ability of two currently popular statistical approaches to metabolomics data (PCA and BL-SOM) to analyze, visualize and interpret our results. To our knowledge, this is the first time WGCNA has been applied to a metabolomics dataset.

## Results

### Non-targeted NMR Provides a High-throughput View of the Tomato Fruit Metabolome

For this proof of concept study, we chose a small number of closely related tomato varieties that exhibited significant phenotypic variation to demonstrate the utility of WGCNA to explain metabolomic data. As mentioned above, a hypomorphic allele of *LeMADS-RIN* is in widespread use by tomato breeders to prolong shelf life [34]. This usage implies acceptance by producers, consumers and other stakeholders of the *rin* effect on tomato fruit composition [35]. The *rin* mutation has been introgressed into heirloom varieties (Ailsa Craig [33]), modern breeding lines (NC1rinEC) and commercialized varieties (Mountain Crest [36]). A phenocopy of the *rin* mutant was created to verify identification of the *LeMADS-RIN* gene as responsible for the *rin* mutation [33]. Thus, using six tomato accessions, we could examine the effect of a single gene mutation with strong phenotypic effect in two genetic backgrounds representing different levels of genetic improvement (heirloom versus modern cultivars; Table 1). To further simplify our analyses, we used methanolic extracts from whole, mature fruit grown in a greenhouse to represent a tomato fruit metabolome relevant to human consumption.

**Table 1:** Study panel for tomato fruit metabolomic profiling.

Abbreviation	Description	Source	Note	Fruit at Maturity (color, firmness)
AC	Ailsa Craig (*Rin/Rin*)	Tomato Genetics Resource Center	Heirloom variety	Fully ripe (red, soft)
AC *rin*	Ailsa Craig (*rin/rin*)	Tomato Genetics Resource Center	Heirloom variety with isogenic mutation	Unripe (green, hard)
AC tg *rin*	Ailsa Craig (antisense *LeMADS-rin*)	Ref #33	Heirloom variety with transgenic construct	Incompletely ripe (some ripening due to leaky gene silencing)
NC	NC84173 (*Rin/Rin*)	Ref #36	Modern breeding line	Fully ripe (red, soft)
NC *rin*	NC1rinEC (*rin/rin)*	Ref #36	Modern breeding line (mutant)	Unripe (yellow, hard)
NC F1	NC84173× NC1rinEC (cv. Mountain Crest)	Ref #36	Commercial hybrid with extended shelf life	Incompletely ripe (red, reduced softening)

The NMR dataset was constructed by individually measuring the spectra of methanolic extracts from each of 60 fruits (ten separate fruits per genotype), thereby providing information on the intrinsic biological variation within the samples. Compound identification utilized 1D and 2D spectroscopic techniques to establish through-bond connectivities, and the concentrations of each compound was determined by manual profiling against the spectra of more than 150 standard compounds. This produced a list of 46 metabolites that accounted for most of the NMR intensities, although a significant number of important resonances remain unidentified and could not be profiled. As expected, the use of water-methanol extracts resulted in appreciable concentrations of sugars and acids, which are important biomarkers for flavor, nutrition and food quality. Their inclusion, however, may have also limited the dynamic range of the NMR spectra and obscured other signals [37]. WGCNA is especially adept at combining multiple disparate datasets. In this proof-of-concept study, however, we chose to focus on the metabolite concentrations determined by the profiled NMR analyses, which produces annotated PCA, BL-SOM and WGCNA plots that are extremely information-rich, and provide a better understanding of what might be expected from an analysis with a greater number of variables.

### Principal component analysis clusters tomato fruit by genetic background and phenotype, but does not provide fine-scale information about the metabolome

Principal components analysis (PCA) is a technique whereby the behavior of hundreds or thousands of variables are summarized by a small number of orthogonal "principal components," which are linear combinations of the original variables. The first principal component describes the dimension that displays the greatest variation in the dataset; the second principal component describes the dimension that displays the second greatest variation, *etc*. The first two or three principal components usually describe the majority of the variation seen in the entire dataset. PCA allows the user to view the relative similarity of different individuals along principal components while associating each principal component with a set of original variables. PCA reveals the relative similarity among samples in an unbiased fashion. Although this is an extremely useful tool for the initial exploration of multidimensional data, it is not particularly effective at clustering or classifying data [38], [39]. While PCA score and loading plots often do indicate which features dominate differences among sample types, there is no further explanation. PCA does not inform the user of the larger reasons that underlie differences between sample types, nor does it provide a route to investigate specific differences in more detail.

**Figure 1:**
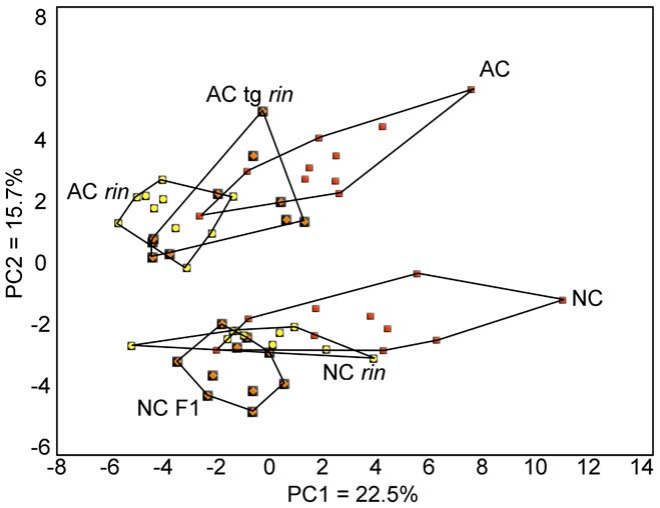
Principal component analysis (PCA) of metabolic profiles of whole tomato fruit. Six tomato genotypes from two genetic backgrounds were analyzed by PCA using 46 NMR-profiled metabolites. Within each background (AC, an heirloom variety; NC, modern production varieties), variation existed at the *Rin* locus such that one fully ripening type (red squares), one partially ripening type (orange diamonds), and one non-ripening type (yellow circles) existed. Ten individual fruits were profiled per genotype.

PCA of non-targeted NMR profiles grouped the six genotypes into two primary clusters with clear separation by genetic background (Figure 1). Principal component 1 explained 25.2 % of the variation in the data and roughly separated genotypes based on ripening phenotype and *Rin* allele status. This component was associated positively with formate, threonine, glutamate, phenylalanine, leucine, GABA, τ-methylhistidine, tyrosine and NADP (loading values >0.20) and negatively with malic acid (loading value <− 0.10). While principal component 1 separated the three varieties in the AC background by apparent degree of ripeness, an explanation for the relative positions of the three NC varieties was not as apparent. Principal component 2 was positively associated with this difference between AC and NC backgrounds and explained 15.7 % of the variation in the data. This component was associated positively with indole-3-acetic acid, malic acid, an incompletely identified sterol, chlorogenic acid, citrate, and coumaric acid (loading values >0.20) and negatively with aspartate, indole, glucose and cytidine (loading values <−0.20).

### Batch learning self-organizing map (BL-SOM) clusters metabolites by expression pattern but does not illustrate relationships among genotypes

**Figure 2:**
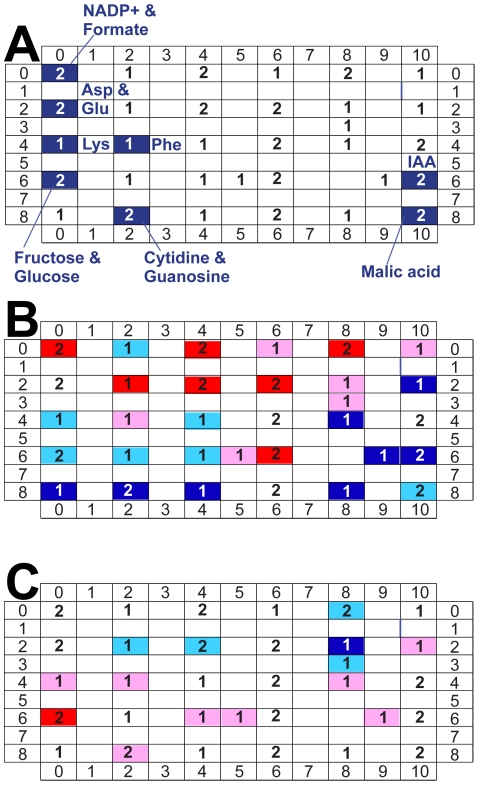
Batch Learning Self Organizing Map (BL-SOM) analysis of metabolic profiles of whole tomato fruit. Six tomato genotypes from two genetic backgrounds were analyzed by BL-SOM using 46 NMR-profiled metabolites. Metabolites were clustered by expression patterns among six genotypes, with highly similar metabolites appearing in the same cells and similar metabolites appearing in adjacent cells. **A. BL-SOM Lattice.** Numbers indicate how many metabolites are contained within each cell. Metabolites that were clustered in the “blue” module of WGCNA are labeled to facilitate comparison. A complete representation of metabolite locations within the BL-SOM analysis is presented as Figure S1. **B. Comparison of AC sample #1 with population mean values**. The metabolome of a single wild type AC fruit was compared with the population mean values using BL-SOM. Red highlighting indicates cells where one or more metabolites are more abundant in AC than the population (red: greater than one standard deviation above the mean; pink: less than one standard deviation above the mean). Blue highlighting indicates where one or more metabolites were less abundant in AC than the population (blue: more than one standard deviation less than the population mean; turquoise: less than one standard deviation less than the population mean). The four cells without highlighting were not different in the comparison. **C. Comparison of AC sample #2 with NC sample #5.** The metabolomes of two single fruit were compared with each other. Nine cells contained metabolites more abundant in AC than NC; five cells contained metabolites less abundant in AC than NC.

BL-SOM clusters metabolites in a two-dimensional matrix according to relative similarities in expression patterns and has been used extensively in plant metabolomic studies [7], [40], [41], [42]. Metabolites with similar expression patterns are located in adjacent cells in the matrix and metabolites with nearly identical expression patterns share the same cell. This method makes comparisons between any two samples easy to visualize. One can also easily compare one sample versus the mean population values. BL-SOM was used to analyze our tomato data set [43]. Based on the relationships detected and the rules of the program, lysine was placed adjacent to phenylalanine, while fructose and glucose were collocated, as were aspartate and glutamate (Figure 2A, Figure S1). A heat map was generated to describe the metabolome of a wild type AC fruit relative to the mean values for the complete data set; thirteen clustered metabolites were more abundant in AC than the population mean, while fifteen were less abundant (Figure 2B). A relative comparison was made between one of the AC and NC samples; nine clustered metabolites were more abundant in AC sample #2 than NC sample #5, while five were less abundant (Figure 2C). However, there is no direct way to compare genotypes represented by multiple replicate samples, or to make higher order comparisons. Together, PCA and BL-SOM indicate which metabolites share similar expression patterns and which vary the most among genotypes, but provide limited insight to understand higher order relationships within a complex data set.

### Weighted correlation network analysis clusters metabolites by expression pattern, identifies highly-connected “hubs” and associates specific modules with genetic and phenotypic traits

WGCNA is a correlation-based method that describes and visualizes networks of data points, whether they are gene expression estimates, metabolite concentrations or other phenotypic data [6]. The actual connectivity of features (topology) of the network is indicated by their position in a dendrogram or other network diagram. Features are clustered into co-expressed "modules" so that one can easily appreciate the complete dataset. Module assignment in WGCNA is a flexible process that permits the user to influence the minimum number of features contained in each module, and therefore the total number of modules identified. Each module is obtained through semi-automated pruning of the dendrogram and is notated by a unique color. Summarizing a network with a limited number of modules can reduce the complexity of a dataset from hundreds or thousands of metabolites or genes to a far smaller number of modules, which can be analyzed with greater statistical power using univariate or multivariate statistics. This overall approach permits broad-scale statistical analysis of feature clusters while retaining the fine-scale relationships for further analyses. This powerful feature of WGCNA allows the user to explore sample-dependent differences at multiple scales, possibly offering explanations for any observed differences.

### Network statistics based on WGCNA

**Figure 3:**
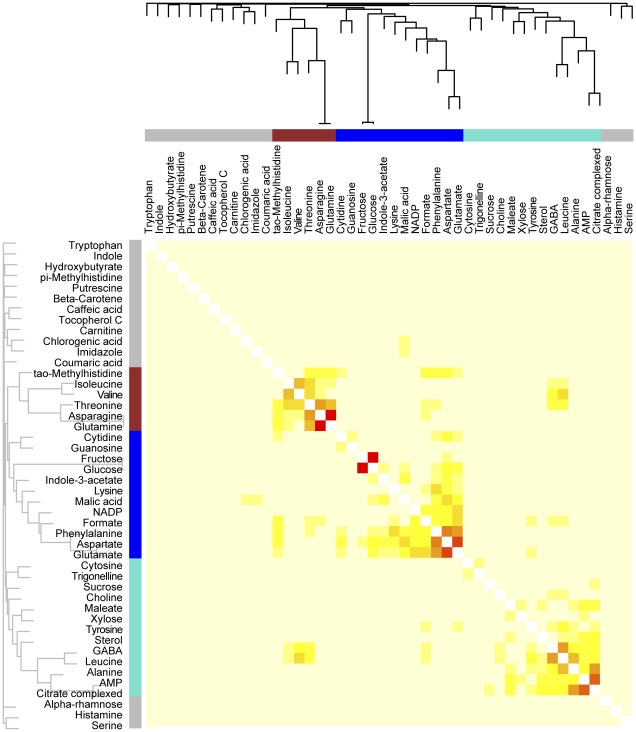
Weighted correlation network analysis (WGCNA) of metabolic profiles of whole tomato fruit. Six tomato genotypes from two genetic backgrounds were analyzed by WGCNA using 46 NMR-profiled metabolites. Metabolites were clustered by expression patterns as represented by the dendrogram and correlation heat map. Clusters of like-regulated metabolites are referred to as modules by color (red, blue, turquoise). Metabolites that could not be assigned to a module are labeled gray. In the heat map, intensity of red coloring indicates strength of correlation between pairs of metabolites on a linear scale.

WGCNA supports the assembly of both signed and unsigned networks. Here, we constructed unsigned networks using the 46 metabolite data set, which co-localize both positively and negatively correlated metabolites into three modules (Figure 3). The WGCNA package additionally provides easy quantification of several network statistics, or indices [5], [44]. In a weighted correlation network, connectivity equals the sum of connection strengths between a node and all of its neighbors, which has been associated with essentiality in protein and metabolic networks [45], [46]. Additionally, highly connected hubs may play a disproportionate role either in influencing the expression patterns of other nodes in the network, or alternatively may act as "sentries," communicating changes that occur elsewhere in the network. Scaled connectivity indicates the connectivity of a given node relative to the most connected node within the same module. The maximum adjacency ratio is related to connectivity; low values indicate nodes with many, weak connections to their neighbors, while high values indicate nodes with few, strong connections to their neighbors. In some situations, the maximum adjacency ratio may be more effective than connectivity to identify important hub features [5]. The clustering coefficient indicates the local density of a network, or the extent to which a node's neighbors are all strongly connected to each other. Other statistics are used to describe modules instead of individual nodes. Network density describes how tightly co-expressed a set of nodes within a module are, while centralization and heterogeneity describe the extent to which nodes within a given module differ in connectivity. Network heterogeneity describes the variation in connectivity within a module while centralization describes the extent to which a network contains many nodes that connect to a central hub node, but do not connect to their neighbors [5].

### Module analysis based on WGCNA

**Figure 4:**
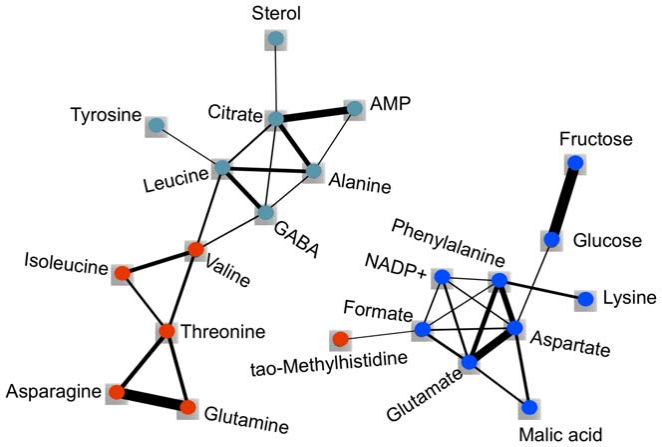
WGCNA of whole tomato fruit metabolic profiles as represented by node and edge graph. 22 of 46 NMR-profiled metabolites were clustered into three modules (red, blue, turquoise); remaining metabolites were not assigned to any module (color coded as gray). Connection strength is represented by edge width (edges <0.10 omitted). The topological overlap measure from the WGCNA was displayed using Cytoscape to illustrate the network assembled from the 22 metabolites.

Module and metabolite-specific network statistics were calculated for this network (Tables 2 and 3). The turquoise and blue modules were the largest modules, containing 13 and 12 compounds respectively, while the red module contained 6 (Figure 3, Table 2). Fifteen metabolites were not assigned to any module, and were labeled with the color gray. All three modules are similar in centralization, while the red module is denser and less heterogeneous than the blue and turquoise modules; these features can easily be observed when the network is displayed using Cytoscape (Figure 4) [47]. Aspartate has the highest connectivity in the dataset (1.66) and, logically, the highest scaled connectivity within its module (1.00; Table 3). It surpasses all other metabolites in its number of connections, many of which are strong and link shared neighbors. Sterol, which has a single, very weak connection, has the lowest connectivity among the three modules (0.45). Fructose has the highest maximum adjacency ratio among all metabolites (0.26), which is reflected in its single, yet extremely strong connection to glucose. This connection is consistent with the precursor/product roles of glucose and fructose in glycolysis and gluconeogenesis [48]. Similar precursor/product or product/co-factor relationships were recognized by the WGCNA between isoleucine and threonine, leucine and GABA, and alanine and AMP, as evidenced by their high connectivity within the network (Figure 4) [48]. Formate has one of the lowest maximum adjacency ratios in the dataset (0.08) and has many weak connections. The lowest maximum adjacency ratio within the turquoise, blue, and red modules (0.05) belongs to tyrosine, which has a single, very weak connection. NADP+ has the highest clustering coefficient (0.11) due to the dense connections among all of its neighbors. Glucose has the lowest clustering coefficient within the three modules (0.05), which is reflected in its connection to two completely unconnected nodes.

**Table 2:** Network statistics for modules.

Module	Node Count	Density	Centralization	Heterogeneity
Turquoise	13	0.04	0.06	0.67
Blue	12	0.06	0.08	0.51
Red	6	0.12	0.06	0.34

Node count indicates the number of features assigned to each module. Density describes how tightly interconnected the features within each module are. Centralization indicates the extent to which modules contain hubs with much higher connectivity than their neighbors. Heterogeneity describes the variation in connection strength among features in each module. Metabolite features that were not assigned to a module are not included in this table.

**Table 3:** Network statistics for the three most connected hub features in each module.

Feature	Module	Whole Network Connectivity	Scaled Connectivity	Clustering Coefficient	Maximum Adjacency Ratio
Leucine *	Turquoise	1.32	0.80	0.05	0.11
Citrate *	Turquoise	1.27	0.77	0.06	0.16
GABA *	Turquoise	1.13	0.68	0.06	0.10
Aspartate *	Blue	1.66	1.00	0.06	0.16
Glutamate *	Blue	1.54	0.93	0.07	0.15
Phenylalanine *	Blue	1.36	0.82	0.07	0.13
Threonine *	Red	1.31	0.79	0.06	0.12
Asparagine	Red	1.05	0.64	0.07	0.22
Glutamine	Red	0.99	0.60	0.08	0.22

Whole network connectivity indicates the extent to which a node is connected to many other nodes, while scaled connectivity is a relative measure of connectivity within each module. Cluster coefficient reflects the extent to which the neighbors of a node are all connected to each other. Maximum adjacency ratio indicates whether a high connectivity is due to few, strong connections or many, weak connections. Asterisks indicate features that were also identified as significant by PCA.

**Figure 5:**
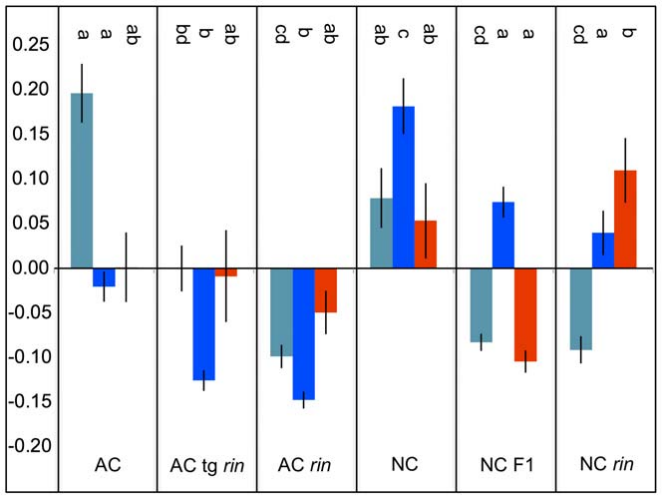
Association of WGCNA module eigenmetabolites with tomato genotypes. ANOVA was used to compare the typical expression patterns (eigenmetabolites) of each module. Significant differences in eigenmetabolites among genotypes within for each module are indicated by letters (at Bonferonni-adjusted threshold of 0.0167). Metabolites in the blue module were more highly expressed in the NC than the AC background. Metabolites in both the blue and turquoise modules were more highly expressed in fully-ripe relative to partially-ripe and unripe fruit. Interestingly, the metabolites in the red module were high in both NC and NC *rin*, but not in the NC F1 hybrid.

One of the most common challenges in systems biology experiments is that of multiple testing. It is extremely common to have very few observations on hundreds to tens of thousands of different entities (e.g. metabolites, genes). WGCNA addresses this issue by allowing the user to investigate associations among specific network nodes or clusters with other factors, such as genetic background or the impact of a mutation. For example, instead of searching for correlations between a given factor or trait (e.g. ripeness) and thousands of genes in a dataset, attention could be focused only on the most highly-connected “hub” genes that might be expected to play the most influential regulatory roles. Alternatively, external traits can be compared to the typical expression pattern (an “eigengene”, or analogously an “eigenmetabolite”) of putatively co-regulated modules instead of to every constituent molecule individually. In our analysis, the turquoise module is positively associated with wild-type fruit ripening (and the presence of functional *Rin* alleles) (Figure 5). The blue module shares this pattern and is additionally positively associated with the difference in genetic background (AC *versus* NC), possibly indicating which metabolites prevented principal component 1 of the PCA from completely predicting ripeness. By estimating a set of *eigenmetabolites*, WGCNA allows the user to apply commonly used and well-understood statistical approaches such as ANOVA to investigate specific hypothesis within the data, by limiting the number of necessary comparisons that are required to query the entire data set.

## Discussion

A new emphasis on the thoughtful use and adoption of statistical analyses is required in order for the biological sciences to keep pace with the increasing dominance of complex and highly multivariate systems biology data. Here, we compare the relative utility of two common and one novel statistical approach to investigate a non-targeted metabolomic profiling dataset. While our present data set contains only six related varieties and 46 compounds, we assert that this is an adequate test case to evaluate different statistical methods and draw attention to the need for more descriptive and powerful statistics in the analysis of metabolomics data. PCA revealed relative variation among the six genotypes but was only able to separate genotypes based on the major sources of variation. While the differences between the AC and NC genetic backgrounds were identified and visualized, the potentially more interesting differences associated with *Rin* allele state and ripening phenotype were not. For example, while PCA demonstrated that AC tg *rin* is intermediate between AC and AC *rin*, as would be expect by the gross phenotype, PCA did not allow us to ask if there are fine scale differences in phenotypes caused by two expression states for *Rin* as a required factor for ripening. Additionally, while NC and NC *rin* are the parents of NC F1, which has an intermediate ripening phenotype to its parents, PCA did not assign these three varieties as we would have predicted. PCA placed NC *rin* as intermediate between NC and NC F1, where NC *rin* was nearly contained within NC, in spite of the fact that NC was fully ripe, NC *rin* was unripe, and their hybrid was intermediate. This is a fundamental limitation of PCA that extends even to the major sources of variation. The few metabolites that explain most of the variation among genotypes are often obvious, *e.g.* with loading values well above +0.1 or below −0.1, but the identification of other, potentially significant differences are difficult as there is little further discrimination among features. Additional variables are presumed to be unimportant because they explain little of the total variation, but this cannot be confirmed empirically if such secondary candidates cannot be identified. Similarly, as PCA plots are dominated by the influence of those metabolites that best describe the overall variation, adding additional layers of information may not enrich the analysis.

BL-SOM rapidly allows the user to visualize relationships between metabolites in a test population, using a lattice to organize the results [43]. This method organizes like regulated metabolites into cells of the lattice, where heat maps can then be generated to illustrate differences between single samples and the population mean or any two samples (Figure 2). BL-SOM does not offer much flexibility for analysis, nor does it facilitate making every pair-wise comparison between samples and sample types within a complex dataset.

WGCNA differs fundamentally from the methods mentioned previously by defining a network that continuously links all variables and then clusters the most highly co-expressed variables in flexibly defined modules. Explicitly defining all of the relationships between metabolites at multiple scales facilitates additional comparisons in a way that the lattice display of BL-SOM does not. WGCNA can be used to create both signed networks, which separate positively and negatively correlated nodes into separate modules and also unsigned networks, which assess correlations by their absolute values. We chose to use unsigned networks in this study, which is consistent with the coordinated changes in the tomato metabolome that occur during ripening [20], [22]. Grouping features into modules has several advantages. First, condensing a very large network into a small number of modules or, alternately, hub nodes, allows external traits to be compared to a limited number of variables, providing a solution to the multiple testing problem. This is also consistent with the biology of ripening, where many structural genes are regulated by small number of key regulators such as *Rin* [*20*]. Second, module construction provides a means by which the roles of unidentified or poorly characterized molecules can be inferred from their better-annotated neighbors. The identification of co-regulated modules helps to annotate the results from systems biology scale experiments, adding valuable biological information. Third, since the influence of minor variables is not masked by the most dramatic differences in terms of absolute scale, as occurs in PCA, WGCNA allows the combining of disparate datasets. Preliminary analysis of other metabolomics data collected from these tomato fruits using liquid chromatography/mass spectrometry suggests that WGCNA can combine thousands of compounds that vary over many logs of abundance in a highly meaningful way, including the clustering of precursor and product ions of provisionally identified compounds (DiLeo, Strahan and Hoekenga, unpublished observations). This provides further evidence that WGCNA is an appropriate method for metabolomic fingerprinting and profiling data beyond the analysis of microarray and physiological trait data for which it was created. Finally, WGCNA allows the topology and dynamics of the molecular network itself to be studied, providing a truly systems perspective. The corresponding molecular networks of various biological entities can be compared at multiple scales, assessing both the presence of major, high-level reorganization and fine-scale differences. While BL-SOM also groups metabolites into co-expressed clusters, this method does not define the relative similarity among metabolites, making the assignment of metabolites to different modules difficult. Also, unlike WGCNA, BL-SOM does not allow the user to compare relative differences among different classes of samples, nor does it provide network statistics. In addition to the statistics described here, WGCNA can also be used to analyze causality in a network, using genetic markers or other data [5], [44], [49].

While it is not novel to propose a method of generating molecular co-expression networks, few approaches provide such a complete network investigation toolkit as WGCNA, particularly in the straightforward generation of network statistics, the flexible yet objective delineation of modules and the ability to compare the typical expression values of a small number of modules or hub molecules to external traits. While the implementation of WGCNA in R is not as straightforward as a standalone program such as SimpleSOM or many software packages that provide for PCA and ANOVA, we believe that the extent to which WGCNA gives insight into and control of data gives it a critical advantage over relying on more common but less powerful methods. While future methods will certainly eclipse WGCNA's effective balance of analytical power and ease of use, it is unlikely that biology will soon return to a state where groundbreaking work can be done with minimal statistical analysis. WGCNA, when incorporated into a thoughtful data analysis plan, can provide not only a rich and multi-perspective view of systems biology data, but also an easy introduction to the R statistical environment, and therefore facilitate data analyses where the investigator has greater flexibility and control over the process of analysis.

### Conclusions

It increasingly appears that much of the low-hanging fruit in crop genetics (*e.g.* non-lethal single genes of major effect) have already been picked [50]. With the advent of affordable, high-throughput systems biology technologies, new statistical approaches are required to fully interrogate the resulting mountains of data. Here, we demonstrate how WGCNA, a powerful and user-friendly approach, can be used to more fully analyze a non-targeted metabolomic profiling dataset than would be possible with more common statistical methods such as PCA and BL-SOM. Specifically, we show how WGCNA can recognize and model systems-level differences in biological networks even where poorly defined phenotypes preclude the use of simple, deductive experimentation. We suggest that this approachable network analysis tool would be extremely useful to biologists who are hoping to condense meaning from large, multidimensional and incompletely annotated datasets.

## Materials and Methods

### Plant material and processing

Tomato varieties were selected from two genetic backgrounds: heirloom Ailsa Craig (AC) and modern accessions from the Mountain Horticultural Crops Research and Extension Center of North Carolina State University (NC; **Table I**). Plants were grown in a greenhouse during the first quarter of 2008 under standard horticultural practices in Ithaca NY. Three AC genotypes with different combinations of *Rin* alleles were included in this study: homozygous wild-type *Rin/Rin* (AC wt), homozygous *rin/rin* mutant (AC *rin*) and transgenic that was homozygous for an antisense *LeMADS-RIN* full-length cDNA construct (AC tg *rin*) [33]. Three NC genotypes with different combinations of *Rin* alleles were included in this study: homozygous wild-type *Rin/Rin* NC84173 (NC), homozygous *rin*/*rin* mutant NC1rinEC (NC *rin*) and the heterozygous *Rin/rin* Mountain Crest (NC F1), which is a commercialized hybrid of NC84173 and NC1rinEC grown in the Mid Atlantic region of the US as a fresh market tomato [36]. Plants were hand pollinated in the greenhouse so that staged fruit could be harvested. Fruit were harvested 15 days past breaker stage except for some AC tg *rin* fruit, which were harvested 7 days past breaker due to difficulties assessing progress through ripening. Wild-type fruit (AC wt and NC wt) and the commercial hybrid (NC F1) were bright red at harvest. AC *rin* fruit were usually green and occasionally yellow. While the AC tg *rin* exhibited full suppression of LeMADS-RIN in the initial description (2002), we observed far less complete gene silencing in the 2008 greenhouse [33]. For this study, AC tg *rin* fruit were generally yellow-orange and occasionally green. NC *rin* fruit were usually yellow, which is consistent with the selection of NC *rin* to have enhanced light dependent ripening responses [36]. Ten fruit from 4–6 plants per genotype were collected according to a standard methodology [51]. Briefly, approximately 10 g segments of whole fruit were flash frozen in N_2 (*l*)_, ground to a fine powder with an Ika Mill, and extracted 3:1 (v/w) in a methanol: formic acid solution (99.875∶0.125 % v/v). These ten fruit per genotype were handled as separate biological replicates and measured individually by NMR.

### Non-targeted NMR metabolic profiling

Methanolic extracts were concentrated using a SpeedVac, then reconstituted to near- natural concentrations by the addition of 500 µL of 400 mM sodium phosphate buffer with a pD value of 6.59, and containing 5 mM TSP for referencing and 2 mM sodium azide to inhibit bacterial growth. Each sample was then centrifuged for five minutes to remove water-insoluble extracts and transferred to a standard 5 mm NMR tube and analyzed using a Varian INOVA 11.7 T (Tesla) NMR MHz, equipped with an inverse-detect probe with Z-PFG. Proton spectra were acquired at 400 MHz and 20 C, using a 1D-NOESY pulse sequence with a 100 ms mixing time and presaturation of the solvent signal during the relaxation times. Standard 90° flip angle pulses were used with a relaxation delay of 5 s, and 256 fid transients with 64K data points were acquired. Proton spectra were apodized with a 0.25 Hz exponential line broadening and zero-filled once.

Selected samples of different tomato genotypes were analyzed in greater depth to assign metabolite compounds. These experiments included 1D-^13^C and 135° DEPT, and gradient versions of 2D homonuclear and heteronuclear experiments. The ^13^C and DEPT experiments, at 100 MHz, were acquired with 25 k or 30 k Hz sweep-widths. The ^13^C spectra were averaged over 10,000 transients, used a relaxation delay of 2 s and a standard 45° flip angle. The DEPT spectra used standard 90° and 135° flip angles, 10,000 transients and a relaxation delay of 1 s. These spectra were processed with a 2.5–5 Hz exponential line-broadening and zero-filled once.

The 2D-NMR studies included gradient enhanced versions of COSY (Correlation Spectroscopy), HSQC (Heteronuclear Single Quantum Correlation) or HMQC (Heteronuclear Multiple Quantum Correlation), HMBC (Heteronuclear Multiple Bond Correlation) (16 Hz coupling) and TOCSY (Total Correlation Spectroscopy). The proton homonuclear COSY and TOCSY (80 ms mixing time) 2D experiments were recorded with spectral widths of 6 k or 8 k Hz in both dimensions, using 4096 points in the directly-detected dimension, and 512 increments in the second dimension; 16 transients per fid were collected with a 2 s delay between scans. The 1H-13C heteronuclear experiments, HSQC (non-edited), HMQC and HMBC (8 Hz and 16 Hz coupling), were recorded using 4096 data points and spectral widths of 6 k or 8 k Hz in the proton dimension (directly-detected). For the carbon (indirectly-detected) dimension, 120–512 increments were acquired with a spectral width of 25 k or 30 k Hz, a 1–2 s delay between scans, and 64–256 transients per fid. The F1 dimension of all heteronuclear spectra were forward linear predicted up to 2 times the number of data points, using the half data set as the basis. Spectra were apodized with a sine or sine-squared function and a shift of 0° or 70°. Assignments were performed using Sparky to analyze through-bond connectivities, and resonance values were compared to those in the literature when available [52], [53], [54], [55], [56]. This raw data was both analyzed directly as a fingerprint (450 features) and manually profiled, revealing 45 metabolites.

Chenomx software (Alberta, CA) was used for baseline correction, spectral binning, and chemical compound profiling of the 1D ^1^H NMR spectra of individual tomato fruits. Raw spectra were initially binned in equally spaced 0.02 ppm increments between 10 ppm and 0.5 ppm, excluding the water peak region between 4.5 and 5 ppm. This enabled the rapid assessment of the overall data quality using PCA. An in-depth analysis of the metabolite concentrations in each fruit was performed using the Chenomx software to manually fit the individual spectra to the Chenomx profile database of compounds, which was augmented by the profiles of ∼20 other standard compounds. The profiles of these standard samples were based on 1D-^1^H spectra acquired at ∼1 mM concentration and used the same buffer as the tomato samples.

### Statistical Analysis

Most statistical analyses were performed in R (version 2.10.1) [10]. NMR profile data were analyzed by principal component analysis (PCA), batch learning self-organizing maps (BL-SOM) and weighted correlations network analysis (WGCNA). Data were autoscaled prior to statistical analyses in order to reduce the dominance of dynamic, high-concentration metabolites. PCA was performed with R package pcaMethods with SVD [10], ANOVA was performed with R package stats and BL-SOM was performed with Simple BL-SOM [43]. Unsigned, weighted correlation networks were produced with R package WGCNA with the default power of six [6], [10].

## Supporting Information

Figure S1 **Annotation of metabolites associated with each cell in BL-SOM.** Six tomato genotypes from two genetic backgrounds were analyzed by BL-SOM using 46 NMR-profiled metabolites. Metabolites were clustered by expression patterns among six genotypes, with highly similar metabolites appearing in the same cells and similar metabolites appearing in adjacent cells. This figure indicates the identity of metabolites contained within each cell of the BL-SOM output (Figure 2). Metabolites are highlighted according to WGCNA module assignment to enhance comparison between analysis methods (Figure 3).
(TIF)
