# Supplementary figures and images for: Weighted Correlation Network Analysis (WGCNA) Applied to the Tomato Fruit Metabolome
Source: PLoS One. 2011 Oct 21;6(10):e26683. doi: 10.1371/journal.pone.0026683 (PMC3198806; doi:10.1371/journal.pone.0026683)

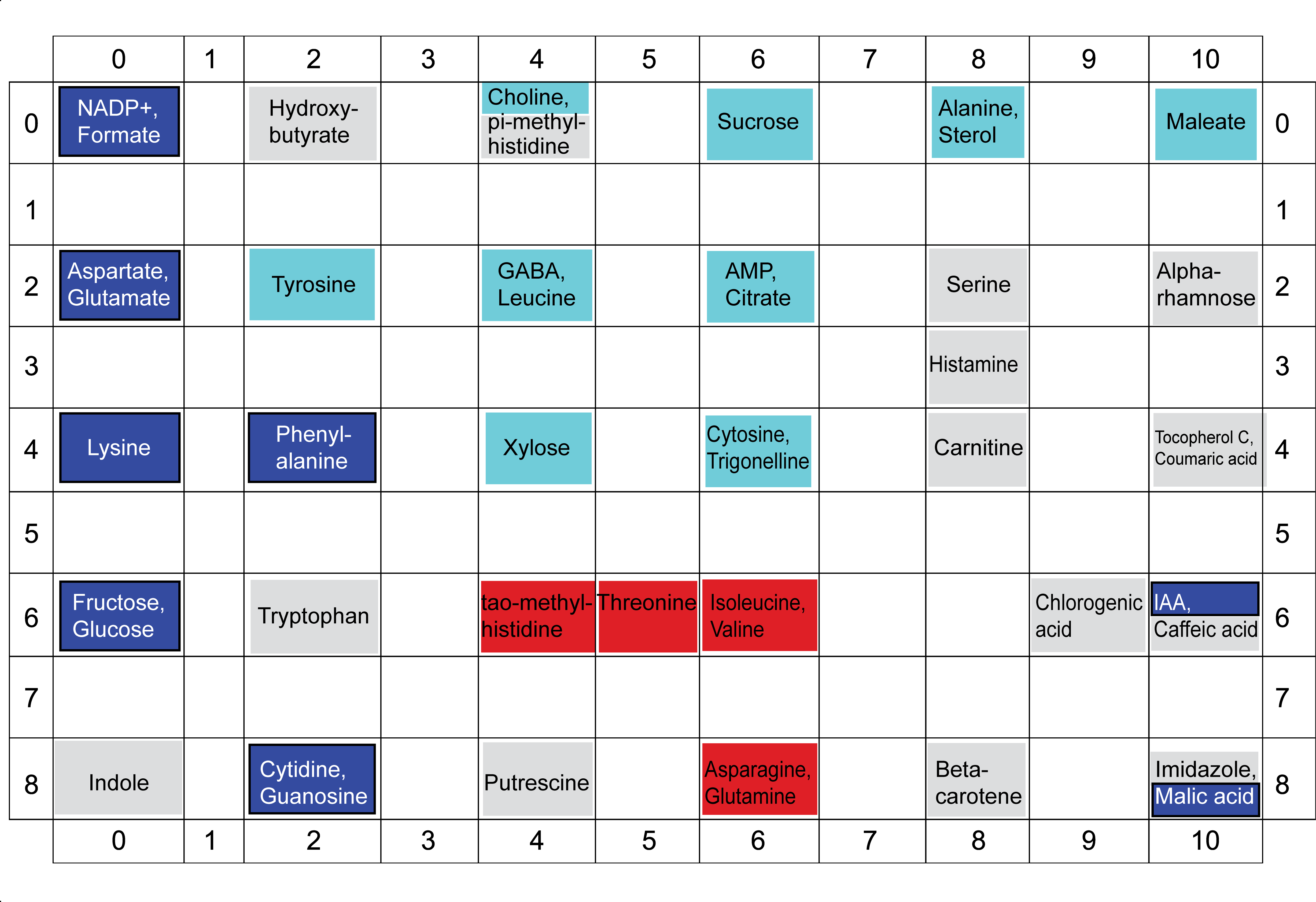

Supplement: Figure S1 — Annotation of metabolites associated with each cell in BL-SOM. Six tomato genotypes from two genetic backgrounds were analyzed by BL-SOM using 46 NMR-profiled metabolites. Metabolites were clustered by expression patterns among six genotypes, with highly similar metabolites appearing in the same cells and similar metabolites appearing in adjacent cells. This figure indicates the identity of metabolites contained within each cell of the BL-SOM output (Figure 2). Metabolites are highlighted according to WGCNA module assignment to enhance comparison between analysis methods (Figure 3). (TIF) [file pone.0026683.s001.tif]
